# High-Precision 3D Printing of Microporous Cochlear Implants for Personalized Local Drug Delivery

**DOI:** 10.3390/jfb14100494

**Published:** 2023-10-03

**Authors:** Aikaterini Isaakidou, Iulian Apachitei, Lidy Elena Fratila-Apachitei, Amir Abbas Zadpoor

**Affiliations:** Department of Biomechanical Engineering, Faculty of Mechanical, Maritime and Materials Engineering, Delft University of Technology (TU Delft), Mekelweg 2, 2628 CD Delft, The Netherlands; i.apachitei@tudelft.nl (I.A.); a.a.zadpoor@tudelft.nl (A.A.Z.)

**Keywords:** hearing loss, porous cochlear implant, two-photon polymerization, surface quality, cytocompatibility

## Abstract

Hearing loss is a highly prevalent multifactorial disorder affecting 20% of the global population. Current treatments using the systemic administration of drugs are therapeutically ineffective due to the anatomy of the cochlea and the existing blood–labyrinth barrier. Local drug delivery systems can ensure therapeutic drug concentrations locally while preventing adverse effects caused by high dosages of systemically administered drugs. Here, we aimed to design, fabricate, and characterize a local drug delivery system for the human cochlea. The design was relevant to the size of the human ear, included two different shapes, and incorporated two different microporous structures acting as reservoirs for drug loading and release. The four cochlear implant designs were printed using the two-photon polymerization (2PP) technique and the IP-Q photoresist. The optimized 2PP process enabled the fabrication of the cochlear implants with great reproducibility and shape fidelity. Rectangular and cylindrical implants featuring cylindrical and tapered tips, respectively, were successfully printed. Their outer dimensions were 0.6 × 0.6 × 2.4 mm^3^ (L × W × H). They incorporated internal porous networks that were printed with high accuracy, yielding pore sizes of 17.88 ± 0.95 μm and 58.15 ± 1.62 μm for the designed values of 20 μm and 60 μm, respectively. The average surface roughness was 1.67 ± 0.24 μm, and the water contact angle was 72.3 ± 3.0°. A high degree of polymerization (~90%) of the IP-Q was identified after printing, and the printed material was cytocompatible with murine macrophages. The cochlear implants designed and 3D printed in this study, featuring relevant sizes for the human ear and tunable internal microporosity, represent a novel approach for personalized treatment of hearing loss through local drug delivery.

## 1. Introduction

Hearing loss is a globally prevalent clinical condition that can be caused by aging (i.e., presbycusis), untreated chronic infections of the inner ear, autoimmune inner ear disease (AIED), conductive hearing loss (CHL), sensorineural hearing loss (SNHL), and noise-induced hearing loss (NIHL) [1]. It currently affects more than 1.5 billion people, representing approximately 20% of the world’s population, and is expected to and affect over 2.5 billion people by 2030 [2,3]. Auditory dysfunctions require different types of treatments depending on the site of malfunction (e.g., ossicular chain, sensory hair cells, auditory nerve). The traditional approach to restoring presbycusis, for example, involves the use of hearing aid devices that amplify sound waves to enhance perception by the patient. However, incomplete hearing restoration and background noise amplification lead to discomfort and long adjustment periods for the patients [4]. Another example is the use of cochlear implants to treat SNHL. The procedure involves the implantation of an electrode to stimulate the cochlear nerve (internal component) as well as the fixation of a wearable device to the skull behind the ear (external component) that communicates with the nerve stimulator. The use of cochlear implants is shown to improve auditory function and language skills in both adults and children [5]. Nonetheless, the invasiveness of the implantation procedure remains a limitation. Despite progress in electrode positioning techniques, the hearing outcome can vary significantly between patients [6]. Moreover, there is a risk of trauma and loss of residual hearing [5]. The systemic administration of drugs, on the other hand, has been clinically practiced in the treatment of vertigo, SNHL, noise hearing loss, and Meniere’s disease [7,8,9]. However, insufficient bioavailability of the drug in the inner ear presents a major challenge. High drug clearance rates from the circulation and minimal uptake through the anterior vestibular artery may result in limited to no hearing enhancement while also creating the risk of organ failure [1]. Local delivery of therapeutic agents (e.g., corticosteroids, antibiotics, aminoglycosides, calcineurin) to the inner ear is, therefore, desirable for the efficient treatment of such conditions and for preventing permanent hearing loss. Nevertheless, the anatomy of the ear hampers the accessibility and controlled release of therapeutics due to the various ear barriers (i.e., the tympanic membrane, the oval and round window, and the blood–labyrinth barrier) that constitute challenges for the delivery of small molecules to the inner ear [3,4,5,6,7]. Current research strategies for local drug delivery include intracochlear infusion of therapeutic agents after cochleostomy, which are promising in terms of sustained long-term drug delivery to the cochlea [10]. In general, intracochlear approaches are highly invasive, and the trade-off between the therapeutic effect and the risks involved (e.g., traumatic electrode placement, loss of residual hearing, and electrode translocation) may not always be favorable [5,6]. Other approaches focus on perforating the natural barrier of the round window membrane to directly reach the intracochlear space [11,12,13]. Related to the latter, dexamethasone-loaded poly(lactic-co-glycolic acid) (PLGA) rod-like extrudates have showcased the possibility of delivering dexamethasone through the round window membrane under in vitro conditions for up to 84 days [13,14]. Silicone microdevices featuring a tip that can be fixed at the round window membrane [12] have been tested in vitro and are shown to be capable of delivering dexamethasone to the inner ear. Nevertheless, drug release rates are limited due to the limited mobility and solubility of the drug in the silicone matrix. Investigations of diffusional mass transport both in vitro and in vivo from silicon-based cochlear implants loaded with dexamethasone showed that drug saturation in the cochlea plays a crucial role in the release rate [15,16].

As is clear from this literature review, there is a need for a local drug delivery system (DDS) to the inner ear that addresses the existing anatomical and functional challenges and limitations. An ideal DDS for the inner ear should (1) increase the bioavailability of the drug in the cochlea, (2) deliver the optimum dose of the drug to the cochlea with the appropriate kinetics, (3) exhibit anatomically relevant sizes and possess suitable mechanical properties, (4) be customizable and be fabricated on-demand to accommodate the patient’s needs, (5) enable the combination of multiple therapeutic agents, (6) decrease the surgical risk and be less invasive than the existing therapies, and (7) be cost-effective.

Recent progress in additive manufacturing (AM) techniques has enabled researchers and pharmaceutical companies to fabricate DDSs with tailored properties, such as intricate geometries at scales relevant to various clinical applications combined with a range of therapeutics [17,18,19,20,21]. For example, stereolithography (SLA), fused deposition modeling (FDM), selective laser sintering (SLS), and binder jetting (BJ) have enabled the fabrication of dosage forms in various shapes, sizes, colors, and flavors [22,23,24,25] with customizable release profiles, a combination of therapeutic agents [26], and pH-responsive drug release behavior [27]. As an ultrahigh-resolution AM technique, two-photon polymerization (2PP) has enabled the fabrication of magnetic steerable helical micro-swimmers for the delivery of cell differentiation factors and neural stimulation [28] and light-responsive micro-swimmers for on-demand local delivery of chemotherapeutic agents [29]. However, AM techniques have not yet been applied for the 3D printing of DDSs for the inner ear.

A high-resolution 3D printing technique enabling the fabrication of millimeter-sized structures is needed to produce implants for the inner ear. Two-photon polymerization is an ultrahigh-resolution AM technique, which is also known and referred to as direct laser writing (DLW), dip-in laser lithography (DiLL), multiphoton polymerization (MPP), 3D laser lithography, and femtosecond laser writing, and is an appropriate choice for this task [30]. Two-photon polymerization involves a nonlinear optical process that takes advantage of the simultaneous absorption of two photons by a molecule [30]. More specifically, a femtosecond laser pulse (100–200 fs) with a mean wavelength in the near-infrared spectrum (~780 nm) simultaneously excites two photons that are absorbed by a photosensitive resin, which is transparent in the infrared spectrum [31]. Photosensitive resins contain photo-initiators that are excited from their ground state and initiate polymerization [32]. This polymerization takes place in small volumes (i.e., voxels) and enables the fabrication of features as small as ~25 nm [33,34]. The intended structure is built by overlapping voxels in a layer-by-layer manner. Recently, this AM technique has been widely studied for biomedical applications (e.g., scaffolds for tissue engineering, microneedles for drug delivery, and 3D micro- and nanotopographies for in vitro systematic studies of cellular processes) [30,35,36,37,38].

Porosity can be used as a storage reservoir for drugs as well as for controlled drug release without the use of additional materials [21,39]. This is of relevance for local drug delivery in various tissues, as it may provide a safer and easier-to-control system compared to the use of biodegradable drug carriers incorporated in the devices. Furthermore, when the location in the tissue is difficult to reach and/or drug bioavailability is limited due to inherent barriers, as in the case of cochlear implants, such systems need to be fabricated with high precision and suitable length scales taking into account the complex anatomy of the site. Therefore, in this study, we investigated for the first time the possibility of designing and fabricating a local DDS for human cochlea featuring internal microporous structures and clinically relevant sizes. To this aim, several different designs have been proposed for the DDS, and 2PP was explored as a method of fabrication. Following optimization of the 2PP conditions to reproducibly and accurately print the designed implants, these were characterized with regard to their morphology, chemistry, surface topography, and wettability. In addition, the cytocompatibility of the printed material was assessed using in vitro assays and immune cells (i.e., murine macrophages).

## 2. Materials and Methods

### 2.1. Implant Design

**Figure 1:**
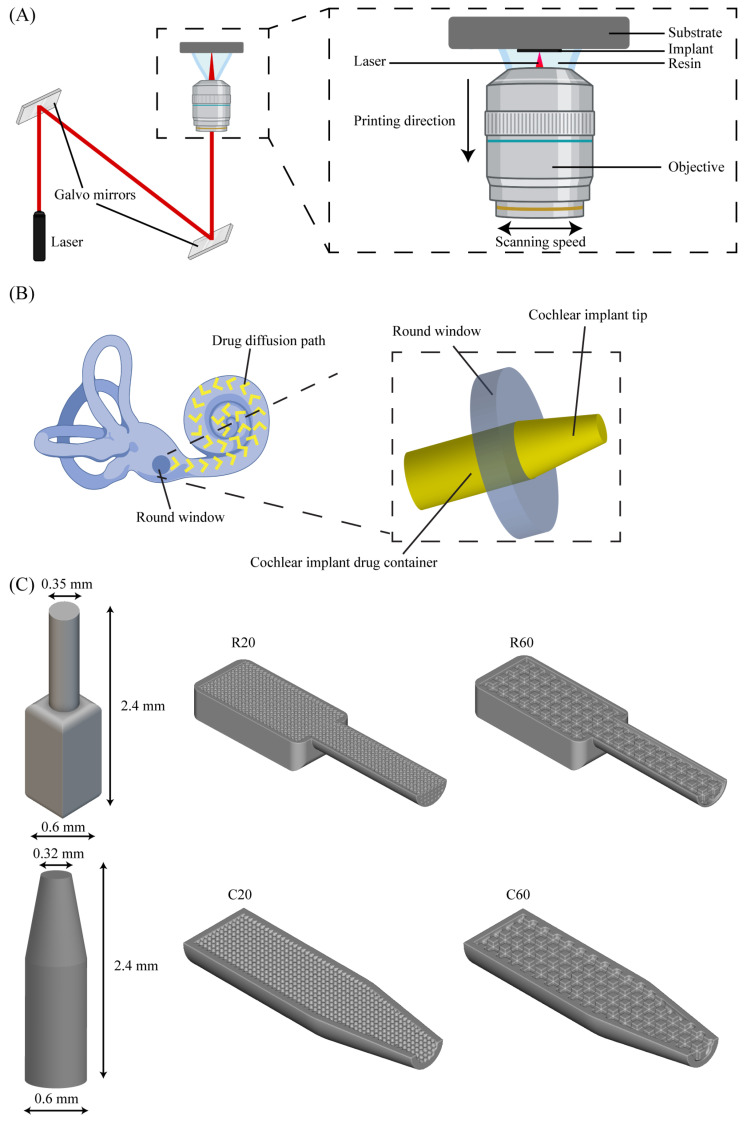
(**A**) The working principle of the 2PP 3D-printing setup, (**B**) a schematic representation of the implant and its positioning in the cochlea, and (**C**) cochlear implant designs and dimensions.

The implants were designed through a workflow that combined two software suites: SolidWorks 2021 (Dassault Systèmes SE, France) and nTopology 2.6 (nTopology Inc., New York, NY, USA). SolidWorks was used to design the outer geometry of the implants, while nTopology was applied for the design of their inner, porous geometry. The final design was assembled in nTopology, where both designs were merged and prepared for printing. The output file was exported as a standard tessellation language (STL) file, which is suitable for 3D printing. The implant design (outer and inner geometries, dimensions) considered the functional requirements and anatomy of the human inner ear (round window). To increase the bioavailability of the locally administered drug and to effectively deliver the drug directly to the cochlea, the design included a main body that serves as a drug reservoir and a tip that penetrates the cochlea through the round window (Figure 1B). Two different implant shapes were considered, namely rectangular implants (R) with a cylindrical tip and cylindrical implants (C) with a tapered tip. The outer designed dimensions of the cochlear implants were 2.4 × 0.6 × 0.6 mm^3^ (L × W × H). An internal porous structure was designed to control the drug release, which consisted of an interconnected network of square pores. Two different pore sizes were included for each shape, namely, 20 and 60 μm, resulting in four different implant designs (i.e., R20, R60, C20, and C60) (Figure 1C). The pore size was chosen based on the results of a preliminary study (as detailed below) and in line with the existing literature on DDSs [40].

### 2.2. Preliminary Study: 3D Printing of Test Structures

The printing process for the cochlear implants began with a preliminary study in which test structures were printed to determine the printing conditions and their relationships with the dimensions of the outer and inner shapes and the porous structure. The test structures included the following: (a) arrays of pillars that were used to determine the printing conditions of high aspect ratio features considering the residual polymerization and its effect on neighboring printed features, (b) cubic unit cells that were used to determine the pore size, and (c) hollow cylindrical structures that enabled us to determine the wall thickness of the cylindrical implants (type C) and the cylindrical tip diameter for the rectangular implants (type R) (Table 1). The results of this preliminary study were then used to define a suitable set of printing parameters for the final designs.

Arrays of 17 pillars with a diameter of *d_d_* = 5 μm, a length of *l* = 80 μm, and an ascending interspace ranging between *i_d_* = 5 μm and 20 μm were designed and printed. The aspect ratio, AR = 16, was chosen according to the manufacturer’s instructions for the solution set for large features to determine suitable printing parameters and to ensure the printability of a complete cochlear implant. The pillar arrays were designed in nTopology (nTopology Inc., New York, NY, USA) and were meshed and imported as an STL file into the proprietary job preparation software of the 2PP printer (i.e., Describe, Nanoscribe, Eggenstein-Leopoldshafen, Germany), where they were prepared for printing. The arrays were printed from the IP-Q resin (Nanoscribe GMbH, Eggenstein-Leopoldshafen, Germany) in the dip-in-laser lithography (DiLL) mode using the microscope z-drive scanning method of the Photonic Professional GT laser lithography system (Nanoscribe GMbH, Eggenstein-Leopoldshafen, Germany) with a scanning speed (*υ*) of 150,000 μm/s, a slicing distance (*s*) of 5 μm, and a hatching distance (*h*) of 1 μm. The effects of three different laser powers (LP_1_ = 25 mW, LP_2_ = 40 mW, and LP_3_ = 50 mW) on the morphology and interspace of the printed pillars were determined. The arrays of pillars were imaged using a scanning electron microscope (SEM) (JSMIT100, JEOL, Akishima, Japan).

Cubic unit cells with an edge length of *l* = 240 μm and strut widths of *s_1_* = 20 μm, *s_2_* = 30 μm, *s_3_* = 40 μm, and *s_4_* = 50 μm were designed using SolidWorks. The geometries were then meshed and imported as an STL file into Describe, where they were prepared for printing. We then printed these with IP-Q resin, in the DiLL mode, and using the microscope z-drive scanning method and the same 2PP equipment. Three different laser powers of LP_1_ = 25 mW, LP_2_ = 40 mW, and LP_3_ = 50 mW were used together with a constant scanning speed of *υ* = 150,000 μm/s, a slicing distance of *s* = 5 μm, and a hatching distance of *h* = 1 μm. Similarly, hollow cylinders with a length of *l* = 240 μm and wall thicknesses of *w_1_* = 100 μm, *w_2_* = 75 μm, *w_3_* = 50 μm, *w_4_* = 40 μm, *w_5_* = 30 μm, and *w_6_* = 20 μm were designed using the same software and were processed and printed under the same printing conditions as described above. The effects of the three different laser powers on the morphology and shape fidelity of the structures were assessed. Following 3D printing, the structures were imaged with the same SEM equipment. All materials and reagents were used as received, unless otherwise specified. 

### 2.3. 3D Printing of Cochlear Implants

The implants were fabricated using the above-described 2PP equipment from the IP-Q photoresist. The 2PP system uses a femtosecond fiber laser with a center wavelength of 780 nm, operating at 80 MHz with a pulse duration of 100 fs. 

All designs were meshed in nTopology (nTopology Inc., New York, NY, USA) and were imported as an STL file into Describe, where a general writing language (GWL) file was produced for printing. The implants were prepared for printing at 2 orientation angles, namely 0° and 90°.

First, square silicon wafer chip substrates (25 × 25 × 0.725 mm^3^) were wiped with acetone (Sigma-Aldrich, Darmstadt, Germany), followed by isopropyl alcohol (IPA) (Sigma-Aldrich, Darmstadt, Germany). Then, they were activated with oxygen plasma for 15 min, followed by silanization for 1 h in a 2% 3-(trimethoxysilyl)propyl methacrylate (Sigma-Aldrich, Darmstadt, Germany) solution in ethanol. Finally, they were rinsed in acetone (Sigma-Aldrich, Darmstadt, Germany) and demineralized water, followed by blow-drying with compressed air.

The galvo writing mode and the dip-in configuration (DiLL) were used to fabricate the samples. Therefore, a droplet of IP-Q photoresist was placed on top of the previously silanized silicon substrates. While printing, the laser beam was focused within the resin through a 10× objective lens with a numerical aperture (NA) of 0.3. The cochlear implants were fabricated with a laser power of LP = 50 mW based on the results of the preliminary study on the test structures. The other parameters were as follows: *υ* = 150,000 μm/s, *s* = 5 μm, and *h* = 1 μm. After printing, the samples were immersed in 1,2-propanediol monomethyl ether acetate (PGMEA) (Sigma-Aldrich, Darmstadt, Germany) for 25 min and then in methoxy-nonafluorobutane C_4_F_9_OCH_3_ (Novec 7100) (Sigma-Aldrich, Darmstadt, Germany) for 30 s. Finally, the samples were dried by blow-drying with compressed air. 

### 2.4. 3D Printing of Samples for IP-Q Characterization

To study the chemical composition, wettability, and cytotoxicity of the printed photocurable IP-Q resin, square blocks of 2.0 × 2.0 × 0.5 mm^3^ (L × W × H) were printed under similar conditions as reported for the implants. Since the size of these samples exceeded the printing field of the 10× objective (i.e., Ø 1000 μm), they were printed in 9 blocks of 705 × 705 × 500 μm^3^ (L × W × H) with a block shear angle of 15° and a block overlap distance of 5 μm in the *xy*-plane. After printing, the samples were processed following the same steps described above for the implants. 

### 2.5. Morphological Characterization

The morphology of the printed cochlear implants was evaluated using SEM. The specimens were, therefore, gold coated using a sputter coater (JFC-1300, JEOL, Akishima, Japan) and imaged under different tilt angles and magnifications with the same SEM equipment as mentioned above. The theoretical porosity of the four implant types was calculated from the digital designs as follows (Table 2):

(1) $$\varphi =\frac{V_{I}-V_{L}}{V_{S}+V_{I}}\times 100\%$$

where *V_I_* is the volume of the solid insert (mm^3^), *V_L_* is the volume of the lattice (mm^3^), *V_S_* is the volume of the implant shell (mm^3^), and *φ* is the porosity of the insert lattice volume (%). 

### 2.6. Surface Topography

The topography of the outer surfaces of the implants was characterized using a non-contact optical profilometer (Profilm3D^®^, Filmetrics Inc., San Diego, CA, USA). The arithmetical mean height (*S*_a_) was determined using the proprietary Filmetrics processing software (ProfilmOnline, San Diego, CA, USA). Each sample was scanned at a minimum of 3 different areas. The mean of the roughness readings corresponded to the areal roughness of that specimen. In total, 3 samples per implant type (i.e., R, C) were scanned. Moreover, line profiles were acquired to determine the average surface roughness (*R_a_*).

### 2.7. Chemical Characterization

The surface chemistry of the square samples after 2PP was assessed using X-ray photoelectron spectroscopy (XPS, Thermo Fisher K-Alpha, Rockford, IL, USA). Three scans were acquired for each square block with an energy step of 0.6 eV using an Al K_α_ source gun with a spot size of 400 μm^2^ in the standard lens mode. Another 3 scans were acquired from points near the square blocks to account for the adventitious carbon contamination on the silicon samples and were used as a charge reference for the XPS spectra correction of IP-Q.

Fourier transform infrared spectroscopy (FTIR) was also performed to identify the functional groups of the IP-Q photoresist before and after printing. The FTIR measurements were conducted using a Nicolet FTIR spectrometer 6700 (Thermo Fisher Scientific, Waltham, MA, USA). The spectra were acquired in the range of 4000–650 cm^−1^ using the attenuated total reflection (ATR) element.

### 2.8. Wettability and Surface Free Energy

The wettability of 2PP printed blocks (5 × 5 × 0.5 mm^3^) was measured using a drop shape analyzer (DSA 100, Kruss, Hamburg, Germany). Droplets (2 μL) of deionized water and diiodomethane (2 μL) were placed on the specimens, and the right and left contact angles of the droplet formed on the substrate were recorded for 2 s at a rate of 5 fps at room temperature (20 °C). The mean contact angle was used in further calculations. The measurements were repeated 5 times. The wettability was expressed as the water contact angle.

The surface free energy was calculated using Young’s equation [41]:

(2) $$\sigma_{SV}=\sigma_{SL}+\sigma_{LV}\times cos\theta$$

where $\sigma_{SV}$ is the interfacial free energy of the solid and the vapor, $\sigma_{SL}$ is the interfacial tension between the liquid and the solid, and $\sigma_{LV}$ is the interfacial free energy of the liquid and the vapor. 

The total surface free energy (SFE) was calculated according to the OWRK method and following the equation [42]:

(3) $$\sigma_{s}=\sigma_{S}^{P}+\sigma_{S}^{D}$$

where $\sigma_{s}^{P}$ is the polar component of the surface energy of the solid, and $\sigma_{s}^{D}$ is the dispersive component of the surface energy of the solid [42,43,44]. 

### 2.9. Cytotoxicity

Murine macrophages (J774A.1, Merck KGaA, Hamburg, Germany) at passage 14 were pre-cultured in 75 cm^2^ flasks (Greiner Bio-One GmbH, Kremsmünster, Austria) at a concentration of 7 × 10^5^ cells per mL with 20 mL of Dulbecco’s Modified Eagle’s medium at 37 °C and 5.0% CO_2_ for 3 days. The 2PP block samples were sterilized in 70% ethanol (Sigma-Aldrich, Hamburg, Germany) (3× for 2 min each) and then rinsed with PBS (10×) (3× for 2 min each). Subsequently, the specimens were seeded with 2.5 × 10^4^ cells per mL in 24-well cell culture plates. 

Macrophage viability was investigated using a live/dead assay with calcein acetoxymethyl (AM) and ethidium homodimer-1 (EthD-1) (ThermoFisher Scientific, Waltham, MA, USA) after 48 h of culture. The cells were rinsed with PBS (10×) and PBS (1×) and were incubated at room temperature in a solution of 0.1 μL/mL calcein AM/PBS (1×) and 1.5 μL/mL EthD-1/PBS (1×) for 30 s. Afterward, the solution was replaced with PBS 1×, and the cells were imaged with a fluorescent microscope (ZOE Fluorescent Cell Imager, BioRad, Hercules, CA, USA). The experiment was performed in triplicate. Cells cultured directly on the well plates and not exposed to the specimens served as the positive controls. 

### 2.10. Image Analysis

Both SEM and fluorescent images were processed with ImageJ 1.47 [45]. In the SEM images, the geometrical characteristics of interest were measured using the Measure command. The fluorescent images were converted to grayscale, followed by a local thresholding step. The cell area was quantified using the Analyze Particle command for both live and dead cells, and the ratio of the dead to the live cells was calculated. Cell viability was calculated as follows:

(4) $$Cell\;viability=\left(1-\frac{Area\;of\;dead\;cells}{Area\;of\;living\;cells}\right)\times 100\%$$

### 2.11. Statistical Analysis

The data are presented as mean ± standard deviation (SD). A Tukey’s multiple comparisons test was performed on the resulting pillar diameter of pillars printed in different conditions. An unpaired *t*-test with Welch’s correction (*p* < 0.05) was used to compare the positive control with the treated group to test macrophage viability on the 2PP printed material. The analyses were performed using Prism 9.5.1 (GraphPad Software, San Diego, CA, USA). 

## 3. Results

### 3.1. 3D Printing of Pillar Arrays

**Figure 2:**
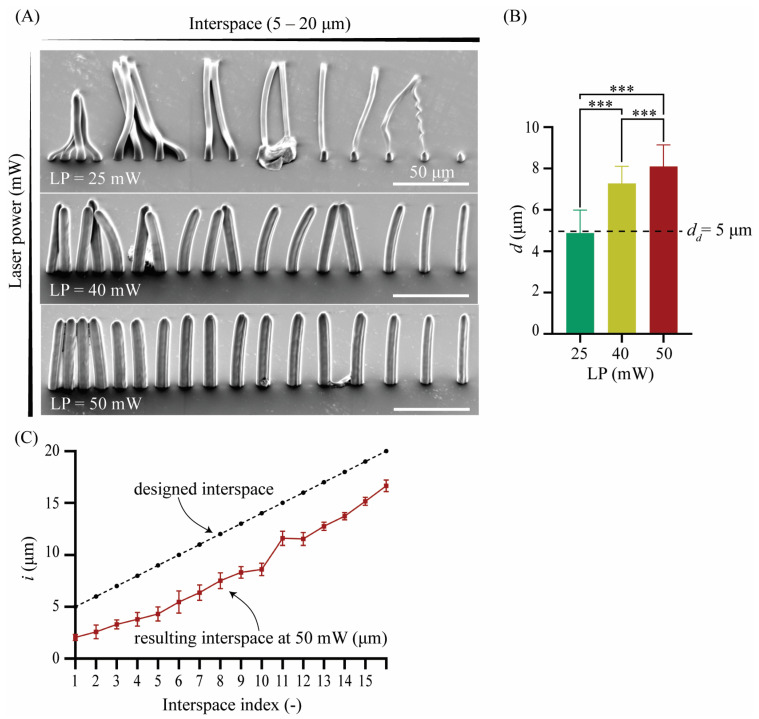
(**A**) The SEM images of the 2PP-printed pillar arrays with a *d_d_* of 5 μm and LP values of 25, 40, and 50 mW (tilt angle = 45°); (**B**) the quantification of the apparent pillar diameter (*d*) and comparison with the designed pillar diameter (*d_d_*) (*** *p* < 0.0001); and (**C**) quantification of the apparent interspace (*i*) for an LP of 50 mW and comparison with the design values.

The SEM images (Figure 2A) showed that, at LP = 25 mW, the resulting pillar diameter was *d* = 4.89 ± 1.10 μm, which is close to the designed value. The actual pillar diameter was larger than the designed one (i.e., *d_d_* = 5 μm) for LP = 40 and 50 mW, namely, *d* = 7.27 ± 0.83 μm and *d* = 8.10 ± 1.03 μm, respectively (Figure 2B). The diameter of the pillars, therefore, increased with the laser power. The differences observed in the printed pillar diameter amongst these 3 different groups printed at 3 different LPs were statistically significant (*p* < 0.001) (Figure 2B). At LP = 25 mW, all the pillars collapsed. At LP = 40 mW, the tips of the pillars appeared bent and fused, even for the larger values of interspacing. However, at LP = 50 mW, the pillars were bent and fused only for the smaller interspacing values. For the same LP (50 mW), the minimum interspace needed for two adjacent pillars to be printed without fusing was *i* = 3.80 ± 0.68 μm, which is, on average, 25% smaller than the smallest designed interspace of *i_1_* = 5 μm (Figure 2C). No minimum interspacing value could be identified for LP = 40 mW and 25 mW since the pillars were either evenly fused for the largest interspacing values or were not printed and appeared collapsed. Altogether, structurally strong and intact pillars of *d* = 8.10 ± 1.03 μm with an interspacing of *i* = 3.80 ± 0.68 μm were printed at LP = 50 mW. The result of this preliminary study indicated the minimum feature size and interspacing that could be feasibly manufactured for such high aspect ratio geometries and laid the groundwork for the following study on 3D porous structures. 

### 3.2. 3D Printing of Cubic Unit Cells and Hollow Cylindrical Structures

**Figure 3:**
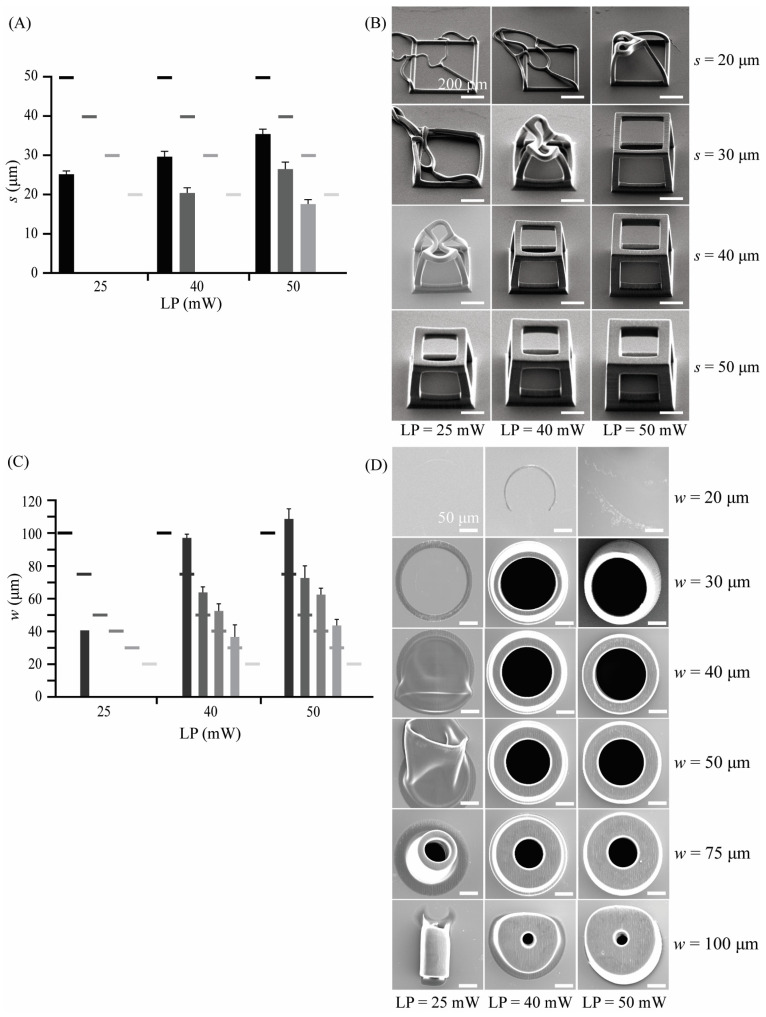
(**A**) The effects of laser power (LP) on the width (*s*) of the struts of the cubic unit cells presented as mean ± standard deviation (*n* = 3) and compared to the design values (grayscale short lines) (scale bar is 200 μm); (**B**) the SEM images (40° tilt angle) used for the assessment of the structural integrity of separate cubic unit cells designed with a strut width of *s* = 20–50 μm for LP = 25, 40, and 50 mW; (**C**) the effects of the laser power (LP) on the wall thickness (*w*) of the hollow cylindrical structures presented as mean ± standard deviation (*n* = 3) and compared to the design values (grayscale short lines) (scale bar is 50 μm); and (**D**) the SEM images (top view) used for the assessment of the structural integrity of the hollow cylindrical structures designed with a wall thickness of *w* = 20–100 μm for LP = 25, 40, and 50 mW.

Cubic unit cells with four different strut widths (*s* = 20 μm, 30 μm, 40 μm, and 50 μm) and hollow cylindrical units with six different wall thicknesses (*w* = 20, 30, 40, 50, 75, and 100 μm) were printed using three different values of the laser power (LP = 25 mW, 40 mW, and 50 mW). The laser power affected the resulting width of the struts and thickness of the walls as well as the general shape of both the cubic unit cells and hollow cylinders (Table 3). Cubic unit cells printed at any LP led to smaller *s* values than the designed ones (Figure 3A). In contrast, hollow cylindrical structures with larger LP values (i.e., LP = 40, 50 mW) exhibited larger *w* values than the designed ones (Figure 3C). The cubic unit cells had resulting strut widths that were 50–70% smaller than designed. The larger the designed strut width, the larger the deviation of the printed width from the designed. The SEM images (Figure 3B) also showed that the cubic unit cells exhibited a truncated shape for most printing conditions. However, this set of prints indicated that a cubic unit cell with a designed strut width of at least *s* = 50 μm could be printed while maintaining the designed shape using the following printing parameters: LP = 50 mW and *υ* = 150,000 μm/s. For hollow cylindrical structures, on the other hand, the actual dimensions of the printed geometries were 20–30% larger than the design values for LP = 40 mW and 30–40% larger than those designed for LP = 50 mW. For both of these LP values and a designed wall thickness of *w* = 100 μm, wall thickness assessment was not possible due to the excessive and irregular shape of the printed structures (Figure 3D). The results of this set of prints revealed that hollow cylindrical specimens yield high-quality prints when printed at LP = 50 mW and *υ* = 150,000 μm/s. They also maintain their overall shape with an overshoot at the wall thickness but can be printed for apparent wall thicknesses (*w*) ranging from ~40 to 100 μm. 

### 3.3. 3D Printing of the Cochlear Implants

**Figure 4:**
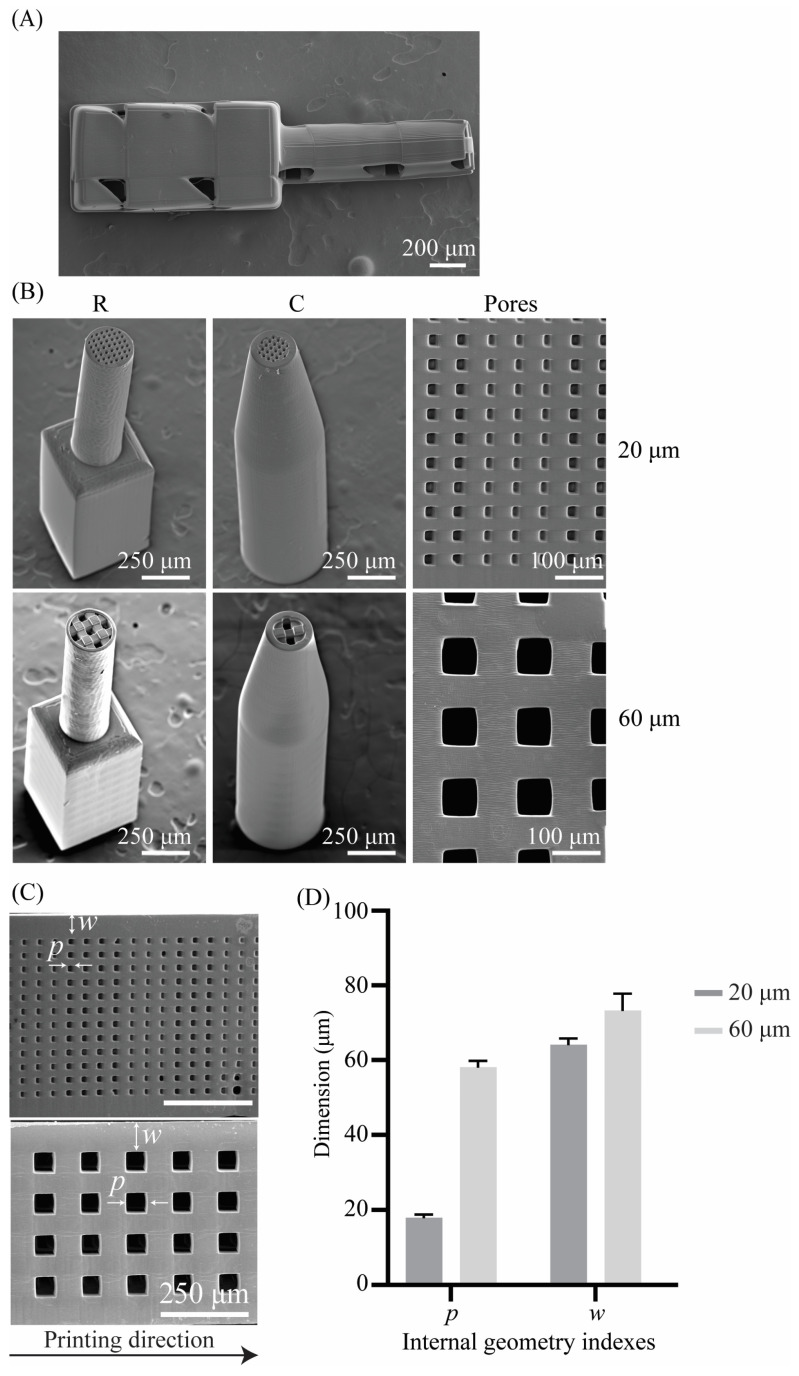
(**A**) A SEM image of a horizontally 2PP-printed R-type cochlear implant (top view); (**B**) SEM images of 2PP-printed porous cochlear implants (tilt angle = 30°) and their internal porous structure and pore size; (**C**) SEM images of the interconnected pore network with pore sizes of 20 μm and 60 μm, where *p* is the pore size and *w* is the wall thickness of the implant; and (**D**) quantification of the pore size (*p*) and wall thickness (*w*). The values are presented as mean ± standard deviation.

The cochlear implants were printed on silicon substrates using two different printing directions, namely, horizontal and vertical. Horizontally printed implants showed signs of defects (Figure 4A). When printed horizontally, the implants exceeded the printing field of the 10× objective (i.e., Ø 1000 μm). Splitting and stitching of the structure in blocks is, therefore, required when preparing the printing job. The defects were located at the stitching sites. Vertically printed samples were printed intact as their base size (i.e., 0.6 × 0.6 mm^2^, W × H) did not exceed the printing field of the 10× objective (Figure 4B). The vertical printing direction and the processing parameters resulting from the preliminary study (i.e., printing of pillar arrays, cubic unit cells, hollow cylinders) enabled the successful printing of the four different designs of the cochlear implants (i.e., R20, R60, C20, C60) (Figure 4B). 

### 3.4. Morphological Characterization

Assessment of the geometry of both interconnected pore networks (i.e., 20 μm and 60 μm) using SEM yielded pore sizes of *p* = 17.88 ± 0.95 μm and *p* = 58.15 ± 1.62 μm, respectively. The wall thickness was also assessed for all the implant types and was *w* = 64.08 ± 1.76 μm for the implant types R20 and C20 and *w* = 72.50 ± 3.38 μm for the implant types R60 and C60 (Table 4). The calculated porosities φ based on Equation (1) for the different cochlear implants were $\varphi_{R60}=52\%$ and $\varphi_{R20}=46\%$ for the R-type implants and $\varphi_{C60}=\varphi_{C20}=50\%$ for the C-type implants. 

### 3.5. Surface Topography

**Figure 5:**
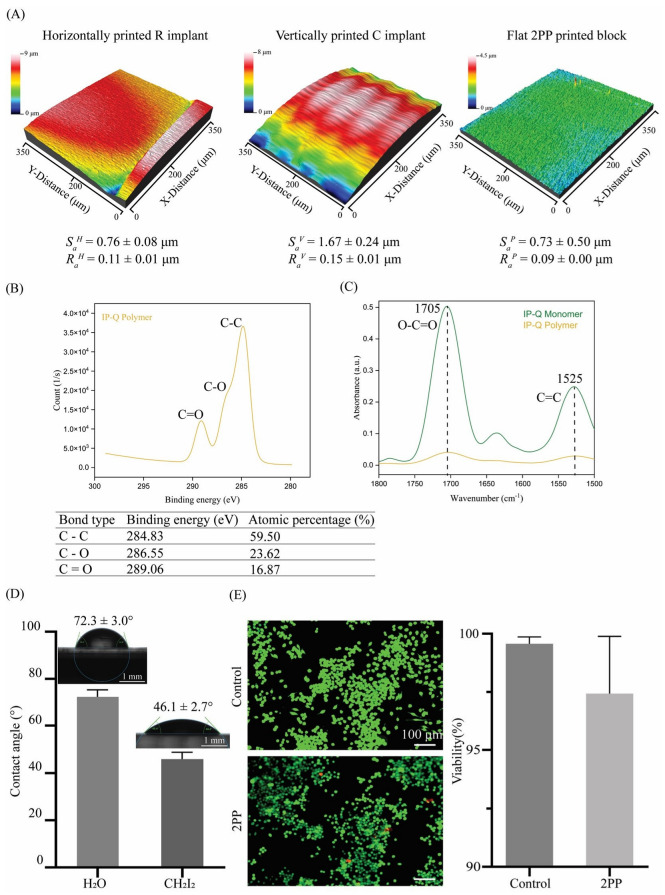
(**A**) The surface roughness measurements (Sa, Ra) for horizontally and vertically printed implants as well as flat 2PP-printed blocks; (**B**) XPS spectra of C 1s peaks in IP-Q after polymerization, and the binding energy and atomic percentage of C 1s series in the 2P-printed IP-Q specimens, (**C**) FTIR spectra in the ATR mode of the IP-Q photoresist (yellow) and polymer after 2PP (green); (**D**) contact angle measurements on flat 2PP-printed IP-Q blocks for water and diiodomethane with the corresponding pictures of the shape of the droplets (scale bar is 1 mm), and the contact angle is presented as mean ± standard deviation; and (**E**) the live/dead staining of J774A.1 macrophages after 48 h of culture on control (i.e., well plate) and on 2PP-printed IP-Q flat block specimens (live: green, red: dead) and the quantification of the macrophage viability (%) for the control and the 2PP-printed specimens (scale bar is 100 μm).

Two surface roughness parameters were measured for the samples used in this study. The average roughness parameter (*R_a_*) was measured over a line profile, whereas the areal average surface roughness (*S_a_*) was measured over a surface. The areal average of the surface roughness of the flat pedestals was *S_a_^p^* = 0.73 ± 0.5 μm, whereas their average surface roughness was *R_a_^P^* = 0.09 ± 0.005 μm. The areal average surface roughness of the implants printed horizontally was *S_a_^H^* = 0.76 ± 0.08 μm, while their average surface roughness was *R_a_^H^* = 0.11 ± 0.01 μm. The areal average of the surface roughness of the implants printed vertically was *S_a_^V^* = 1.67 ± 0.24 μm with an average surface roughness of *R_a_^V^* = 0.15 ± 0.01 μm. The morphology of the vertically printed implants also seems to reveal the layer structure of the 2PP process (Figure 5A).

### 3.6. Chemical Characterization

Peaks with binding energies of 284 eV, 286 eV, and 289 eV are in agreement with C–C, C=C, and C–O bonds, respectively, that have been identified in the XPS spectra of the block samples (Figure 5B). These peaks verify the presence of methacrylate structures [46] in the photocurable resin IP-Q. In the FTIR spectra, a difference in the peak intensity of the carboxyl group (1705 cm^−1^) and carbon double bond (1525 cm^−1^) was observed before and after printing (Figure 5C). More specifically, the intensity of the bonds decreased after printing, indicating double carbon bond depletion as a result of polymerization. The decreases in the intensity of those peaks were ≈ 88% for the carbon double bond at 1525 cm^−1^ and ≈ 92% for the carboxyl group at 1705 cm^−1^. 

### 3.7. Wettability and Surface Free Energy

Water contact angle measurements (*n* = 5) revealed an average water contact angle of $CA_{W}=72.3\;\pm 3.0{}^{\circ}$ and an average contact angle for diiodomethane of $CA_{D}=46.1\;\pm 2.7{}^{\circ}$ (Figure 5D). According to the OWRK model and using the aforementioned contact angle measurements, the total surface free energy was calculated to be SFE=38.1 ±2.3 mN/m, with a polar component of $\sigma_{S}^{P}=1.7\;\pm 1.1\;mN/m$ and a dispersive component of $\sigma_{S}^{D}=36.4\;\pm 1.2mN/m$. 

### 3.8. Cytotoxicity

The live/dead assay using J774A.1 macrophages cultured on the block samples showed high (>95%) viability of the cells after 48 h of culture (Figure 5E). No statistically significant difference in macrophage viability was observed between the control and the test group (*p* = 0.1230). 

## 4. Discussion

We aimed to design, fabricate, and characterize a novel DDS for the inner ear. The results demonstrate the feasibility of using the 2PP method for printing implants with anatomically relevant sizes for the human inner ear. Furthermore, the implants featured internal porous structures to enable the loading of drugs and their controlled release. 

### 4.1. Implant Design and Fabrication 

The fabrication of large-scale structures involves choosing the correct values for several parameters, such as the laser power and orientation angle. An initial investigation of the effects of laser power on the fabrication of pillars with a high aspect ratio (i.e., AR = 16) showed that the height, diameter, and structural integrity of the pillars were strongly dependent on the laser power. The minimum clearance between high-quality pillars with an apparent diameter of ≈8 μm was 3.80 ± 0.68 μm, indicating the highest resolution possible with the setup and material used (i.e., 10× objective, IP-Q resin), which is the only possible combination for the fabrication of large 3D structures using the Nanoscribe system. Using the same configuration, the cubic unit cells that were designed and fabricated to resemble the single pore structure of the implants demonstrated a much smaller strut width (~35 μm), even for the highest quality prints compared to the designed one (~50 μm). The cylindrical hollow units appeared deformed when the wall thickness was 100 μm, even for the highest laser power, due to the large volumetric contraction stress compared to structures with a wall thickness < 100 μm. These results indicate a direct relationship between the printing conditions and the size and strength of the resulting structure. Generally, low laser power results in a low polymerization rate and subsequently in thinner and more fragile structures than higher laser power [47]. A linear relationship has been recently proposed to describe the relationship between the laser power and the thickness of the resulting structure [48]. Moreover, the resulting shape and size of the printed structure are influenced by the shrinkage and buckling that occurs in the post-processing phase [49,50]. In addition, shape and size inaccuracies between the designed and the printed test structures can also be attributed to errors in the stage movements, errors in the tessellated file formats of the designs, and structural deformations of the 2PP-printed structures [51]. The design conceptualization, realization, and 2PP printing of the cochlear implants were based on the results of our preliminary study, in which a wide range of test structures was printed. More specifically, the interconnected porous network was designed with a pore size of 60 μm based on the printing results of the cubic unit cells. The wall thickness was designed to be 50 μm based on the printing results of the hollow cylinders. Finally, the implants were printed vertically so that no stitching would be necessary. In contrast to the test structures, the shape and dimensions of the printed implants substantially deviate from their designs. More specifically, the pore size deviated only 10% from the intended 60 μm. Additionally, the total length and width of the implants were 10% smaller than the designed geometry. Implants with a pore size of 20 μm were successfully realized with similar geometrical deviations from their designs. The difference between the printing of the test structures and the implants can be explained by the residual polymerization, or proximity effect, that occurs in the implants being printed [52]. To put this concept in perspective, for a structure with a height of 240 μm and a slicing distance of 5 μm, 48 layers of material are deposited on top of each other. For a structure that is 10 times larger, the number of layers increases accordingly. The resin, therefore, undergoes many more cycles of exposure to the laser beam, and the heat-initiated polymerization contributes greatly to the final dimensions of the printed structures [53]. Several previous studies have demonstrated that the 2PP process allows for the printing of 3D porous scaffolds with controlled porosity and high precision at the micron and submicron scales [38,54]. In this study, we aimed to incorporate controlled microporosity within millimeter-sized scaffolds suitable for applications as functional cochlear implants. As with the majority of studies, the design of the current study is subject to limitations. The study mainly focuses on the 3D printing feasibility of cochlear implants with an internal porous network consisting of cubic unit cells, and no other type of network has been included. It is known that natural materials develop complex architectures to adapt and function in their environment, with porosity being a crucial factor as it enables filtration, diffusion, and increased permeability for nutrient exchange [55]. The added functional value of porosity has also long been explored in the case of porous scaffolds for various applications [56,57,58]. The use of porous structures specifically for loading and local release of drugs without any additional material/drug carrier is, however, relatively underexplored. Microneedles have been investigated for transdermal delivery of drugs, proving the suitability of such a system [59,60]. The uniqueness of our system lies in the fact that the porous device is printed without the drugs; thus, the printing process is not limited by the drug type or phase. This aspect offers the possibility of creating a versatile structure to be combined with different types of pharmaceuticals and could subsequently be used for different pathologies. It would, therefore, be relevant to investigate the potential of the proposed porosity to control drug delivery and to consider the possibility of another pore shape for the same application. Last, it is important to mention that the fabrication of porous structures with pores in the range of 20–60 μm using the same printing parameters and, thus, within a single step and without block splitting or stitching required could be used to create implants with a combination of different pore sizes to achieve better controllability of the drug release. 

### 4.2. Characterization

The FTIR and XPS results can only qualitatively indicate the degree of conversion of IP-Q. Nevertheless, they verify the methacrylate character of the resin and the change in the carboxylic and double carbon bonds after polymerization. According to the existing literature, the extent of polymerization can be evaluated when quantifying the transformation of double-carbon bonds to single-carbon bonds with respect to the carboxyl group [61]. In our case, both of the peaks indicated the carbon double bond and the carboxyl group decreased in intensity, indicating the involvement of the carboxyl group in the polymerization process. This characteristic is unique to IP-Q compared to other photoresists [62,63,64] and might be related to the fact that IP-Q has been designed for high-speed fabrication. To our knowledge, the polymerization mechanism of the IP-Q material has not yet been fully unraveled. However, the findings of the current study can complement the existing characterization [65]. The contact angle measurements and the resulting SFE indicate a partially hydrophilic character for the 2PP-printed IP-Q. The average surface roughness, *R_a_*, for horizontally and vertically printed implants and flat blocks was in the range of 90–150 nm. The areal (3D) surface roughness, *S_a_*, for the corresponding surfaces was much larger, ranging between 0.730 and 1.670 μm. The difference between those two topographical parameters is related to the fact that for the calculation of *S_a_,* the overall surface is considered, which explains the higher *S_a_* of the C implant types compared to the R implant types. The orientation angle also affected the surface roughness. The higher surface roughness of the vertically printed implant compared to the horizontally printed implant is due to the anisotropic resolution and spatial arrangement of the voxels. The shape of the voxel in the 2PP process resembles an ellipsoid with a higher lateral spatial resolution than the axial resolution. The higher surface roughness at lower orientation angles can also be attributed to the larger slicing distance as compared to the hatching distance. In general, the voxel size and voxel overlap greatly determine the surface finish of a structure, with smaller voxel sizes and a higher degree of overlapping resulting in an improved surface finish. However, the improved surface finish comes at the expense of increased processing and printing times. 

Macrophage viability was comparable between the control polystyrene well plate and the 2PP-printed surfaces, and no statistically significant difference was observed between the two groups. Macrophages survived and proliferated similarly on polystyrene surfaces with a water contact angle of ~55° [66] and on the 2PP surfaces with a water contact angle of ~72°. This indicates the good cytocompatibility of the applied resin and confirms the choice of the resin for the fabrication of such types of implants.

### 4.3. Outlook and Challenges

Hearing preservation through local delivery of drugs, such as antibiotics and steroids [67,68,69], to the cochlea has been recognized as a potentially efficient treatment strategy. However, it remains challenging to locally deliver drugs to the cochlea. A variety of different systems, such as micropumps [67,70], cochlear electrode arrays [71,72], drug-loaded silicone-based ear implants [12] and drug-loaded PLGA nanoparticles [73], have been developed to achieve sustained, controlled delivery and enhance drug bioavailability in the cochlea. The incorporation of porous structures in 3D-printed DDSs to target different pathologies and organs, such as the skin, brain, breast, and cartilage, has been researched by different groups. The structure and dimensions of the implants, on the one hand, and the properties of drugs, on the other hand, influence the release kinetics of drugs both separately and in combination with each other [74]. A general observation is that larger implant surfaces contribute to faster drug release [74]. It has been reported that drug release is affected by the ratio of the pore size of a porous carrier to the size of the drug molecules [75,76]. Moreover, drug loading and release are influenced by the affinity of the carrier and the drug [29]. However, not much information is available regarding the combination of these two effects in the context of cochlear implants. Future research should, therefore, be directed at understanding the interrelations among the 3D printed materials, porous structures of anatomically relevant sizes, and the properties of the drugs that are most likely to be used in conjunction with cochlear implants. Furthermore, the chemical and mechanical properties of such cochlear implants need to be characterized as one of the first attempts to investigate their suitability for application as a DDS. 

## 5. Conclusions

In this study, we successfully designed and fabricated cochlear implants with internal microporous structures using the two-photon polymerization (2PP) technique. Our findings show that the 2PP process is suitable for reliably producing implants with both a rectangular and a cylindrical profile featuring internal square pores of 20 μm and 60 μm. A parametric study on different processing parameters enabled us to identify the settings to achieve the intended feature sizes, surface finish, and structural stability. Moreover, the material used for the fabrication of the cochlear implants (IP-Q) showed a high degree of polymerization and good cytocompatibility for macrophages. Taken together, we demonstrated the feasibility of creating anatomically relevant cochlear implants with precisely controlled and tunable internal microporosity, indicating their potential for localized drug delivery to the human cochlea for personalized treatment of hearing loss.

## Figures and Tables

**Table 1:** The geometry, dimensions, and printing settings used for the fabrication of the test structures, including pillar arrays, cubic unit cells, and hollow cylindrical structures.

	Pillar Array	Cubic Unit Cell	Hollow Cylinder
	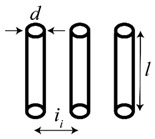	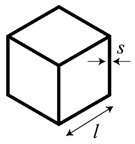	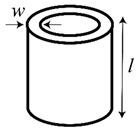
Geometrical characteristics	*l* = 80 μm *d* = 5 μm i_n_ = 5 μm + (*n* − 1), for 1 ≤ *n* ≤ 16	*l* = 240 μm *s* = 20, 30, 40, 50 μm	*l* = 240 μm *w* = 20, 30, 40, 50, 75, 100 μm
2PP printing parameters	LP = 25, 40, 50 mW *υ* = 150,000 μm/s *s* = 5 μm *h* = 1 μm		

**Table 2:** The theoretical porosity φ of the implants based on Equation (1).

	R20	R60	C20	C60
Volume of the implant shell, *V_S_* (mm^3^)	0.15	0.15	0.19	0.19
Volume of the solid insert, *V_I_* (mm^3^)	0.41	0.41	0.33	0.33
Volume of the lattice, *V_L_* (mm^3^)	0.22	0.20	0.16	0.16
Porosity, *φ* (%)	46	52	50	50

**Table 3:** Resulting strut width (*s*) and wall thickness (*w*) compared to the designed values for the three laser power values (LP) (× symbol represents bad-quality prints). The values are presented as mean ± standard deviation (μm).

	Designed *s* (μm)				Designed *w* (μm)					
	20	30	40	50	20	30	40	50	75	100
LP (mW)	Resulting *s* (μm)				Resulting *w* (μm)					
25	×	×	×	25.20 ± 0.84	×	×	×	×	40.64 ± 0.0	×
40	×	×	20.39 ± 1.33	29.65 ± 1.39	×	36.76 ± 7.34	52.59 ± 4.39	63.90 ± 3.46	97.06 ± 2.30	×
50	×	17.57 ± 1.17	26.48 ± 1.79	35.43 ± 1.25	×	43.67 ± 3.71	62.57 ± 4.02	72.70 ± 7.47	108.68 ± 6.13	×

**Table 4:** Morphological parameters of the internal porous network (*p*: pore size) and wall thickness (*w*) of the cochlear implants. The values are presented as mean ± standard deviation (μm).

Parameter	Pore Type	
	20	60
*p*	17.88 ± 0.95	58.15 ± 1.62
*w*	64.08 ± 1.76	72.50 ± 3.38

## Data Availability

The data presented in this study are available on request from the corresponding author(s).
